# Enabling secure and self determined health data sharing and consent management

**DOI:** 10.1038/s41746-025-01945-z

**Published:** 2025-08-30

**Authors:** Cindy Welzel, Max Ostermann, Hannah Louise Smith, Timo Minssen, Toralf Kirsten, Stephen Gilbert

**Affiliations:** 1 Else Kröner Fresenius Center for Digital Health, TUD Dresden University of Technology, Dresden, 01307 Germany; 2 Center for Advanced Studies in Bioscience Innovation Law (CeBIL), University of Copenhagen, Copenhagen, 2300 Denmark; 3 Institute for Medical Informatics, Statistics and Epidemiology, Leipzig University, Leipzig, 04107 Germany

**Keywords:** Health care, Medical research

## Abstract

Digital health tools generate vast sensitive data not only in clinic but also from apps and wearables. Effective consent management builds trust, increasing willingness for data sharing. We present a novel early-stage conceptual framework, reviewing technologies like blockchain, self-sovereign identity, and de-identified tokens for integration into a consent management system to enable tracking of consent and data utilization. This balances data access and privacy to encourage data sharing and improve healthcare.

## Introduction

Digital health tools (DHTs) are increasingly used in healthcare, not only guided by clinicians in hospitals but also driven by commercial entities that have introduced apps and wearables targeted at the health-conscious consumer. These are directly accessible and usable by patients and citizens, independent of their actual clinical needs or only loosely linked to clinical settings^1,2^. In addition, DHTs are increasingly used to help in all aspects of exercise, optimizing sleep, fitness, diet, and even happiness^3,4^. As the range and scope of these tools increase, they continuously increase their ability to detect and interpret intimate details about our surroundings and physiology^3,4^. For example, they track activity, metabolite levels, electrical signals, blood pressure, and oxygen levels^3,4^. These technologies leverage real-time data collection, artificial intelligence (AI), and personalized analytics to potentially enhance patient engagement, improve clinical decision-making, and support preventive care. But despite the numerous advantages of these tools, the question arises as to whether everyone really has an overview of and control over how their health data, generated by these tools, is shared for the direct use in their healthcare and for wider use for research purposes?

The information gathered and processed by wearables and health apps includes highly sensitive personal data, which is generated by individuals in the home care setting with considerable commercial value for many industries^5^. However, the devices and applications on the market are currently not subject to any mandatory quality control and have a correspondingly high potential for misuse. In most cases, users are not meaningfully informed with whom their data is shared, and therefore they cannot object to it being shared. Data and consumer protection organizations therefore regularly warn that users of wearables rarely have control over their own data^6^. A study showed that even the storage duration of the data on their own smartphone can only be adjusted with one of the 13 providers examined, with a minimum storage period of 1 year^6^. Revoking app permissions can only partially restrict the transmission of sensitive data to the provider because apps may still collect certain data through other means, such as cached information, background processes, or alternative permissions. The data is then often stored with large cloud providers, which often means the use of data to train algorithms or the personalized placement of advertisements. None of the apps with an online connection allowed complete offline use or restriction of data traffic^6^. This can lead to security vulnerabilities and cause criminal gateways like identity theft or doxing (publication of highly sensitive data on the internet)^7^.

Nevertheless, DHTs and personally-generated health data (PG-HD) hold great potential to improve treatment (primary use) and research (secondary use), but not automatically override an individual’s ability to control their data and consent to its use^1^. The processing of PG-HD from DHTs must comply with two distinct frameworks: ethical guidelines and legal requirements. The recently updated Declaration of Helsinki and the Nuremberg Code establish informed consent as an ethical foundation for medical research, requiring individuals to be fully informed about all potential outcomes and consequences before granting permission^8^. This allows patients to make informed decisions about their care or understand how their health data is collected, stored, and processed^9^. However, these frameworks primarily address clinical trials and are less applicable to the secondary use of health data from DHTs.

Interacting and partially overlapping with other relevant regulations, such as the European AI Act, the Medical Device Regulation (MDR) and the emerging European Health Data Space (EHDS, Supplementary Table 1)^10–12^, the GDPR establishes the binding legal framework for health data processing^13^. Under Articles 6(1)(a) and 9(2)(a) GDPR, explicit, specific, and informed consent is required where no other legal basis permits the intended processing. Article 89 GDPR, in conjunction with Article 5(1)(b), allows further processing for research purposes under specific safeguards. Different consent models have been proposed to balance research needs with data protection requirements, as specific informed consent before each processing operation may hinder scientific research. One model is broad consent, where individuals agree to future research on their data for up to 5 years and can revoke consent at any time, though critics argue it lacks true informed consent^14,15^. Another model, dynamic consent, allows individuals to provide or withdraw consent via a web or app platform, offering more patient control but potentially leading to “consent fatigue” due to the need for frequent, detailed consent requests^16–19^. A third model, meta consent, combines broad and dynamic consent, allowing individuals to choose how they want to provide consent for past and future data use (Table 1)^20^.

**Table 1:** Overview of Consent Models in Health Data Sharing

Model	Consent timing	User control	Flexibility	Challenges
Broad consent	One-time, upfront	Limited	Low	Lacks granularity, limited reconsent
Dynamic consent	Ongoing, case-by-case	High	High	Consent fatigue, Tech requirements
Meta consent	User-defined (broad or dynamic)	Moderate to high	High	Complexity in user preference management
Standard Health Consent (SHC)	Platform-based, standardized process	High	High	Requires infrastructure and standardization

A recently proposed consent approach—the Standard Health Consent (SHC)^21^ - would combine features of all three consent concepts and would give citizens control over their health data while enabling primary and secondary data sharing through an easy-to-use and standardized consent process. In a platform-based approach the SHC would first dictate the citizen consent preferences for the collection of their clinic- and citizen-generated health data in their personal health record and then the subsequent sharing of this data for primary and secondary use (Table 1)^21^.

But ethical and regulatory frameworks are not enough to give people control over their PG-HD. To address this gap, this paper explores how emerging technologies can be harnessed to provide individuals with meaningful, transparent control over the use and sharing of their PG-HD. In response, we propose an early-stage conceptual and technical framework that combines emerging technical tools, such as blockchain, self-sovereign identity, and de-identified tokens, with a consent management platform to form the foundation of a system that enables verifiable tracking of consent and data use. Our hypothesis is that by enabling better control over and transparency of their data, individuals will be more likely to share their data, which in turn advances medical research and contributes to the common good. Enabling patient control over health data for societal benefit was also acknowledged within the EHDS proposal^22^.

### The willingness of patients and citizens to share their health data

The potential for medical progress through the use of health data, especially PG-HD for big data analytics and AI development, is widely recognized^1,2,21,23^. However, public trust and transparency emerge as crucial determinants of willingness to share such data, alongside regulatory, ethical, and social challenges^23^. While surveys indicate high overall willingness to share health data for research purposes, e.g. 79% of the German population are willing to share anonymized health data for research, with 73% agreeing to long-term use, these findings highlight the nuanced relationship between transparency and trust^23,24^. A preference for active (opt-in) consent mechanism over passive (opt-out) models (88% vs. 12%) was shown^25^. Detailed information about data usage, storage, privacy and data protection significantly boosts willingness to share, as transparency fosters a sense of control, aligns with public expectations and is also key to mitigating perceived risks, such as breaches of confidentiality, misuse, and commercialization^26–28^. Conversely, the opacity of processes, especially regarding the actors involved, undermines trust. A critical shortcoming of existing regulations like the GDPR lies in their failure to reflect public concerns about who can access their data. Citizens prioritize transparency about actors and their roles, indicating that building trust requires closing this transparency gap. While citizens express their comfort at sharing data with healthcare professionals (HCPs), academic researchers, and non-profit organizations, there is notable reluctance toward for-profit and governmental organizations^26,29–36^. Addressing these themes is essential to bridging the gap between public willingness to share PG-HD and the operational needs of research, highlighting the pivotal role of transparency in determining support for health data sharing initiatives.

### Tracking of consent and health data use

We have proposed, based on the research cited above, that it would increase patients’ and citizens’ willingness to share their PG-HD for research purposes if they were able to transparently view and change their consent to the use of their PG-HD at any time as found in the proposed SHC concept^21^. We further propose that if patients and citizens receive timely feedback on the purposes and research projects for which their data is used, this would further increase their willingness to share their PG-HD. To enable this, tracking individuals’ consent and actual uses of anonymized PG-HD is needed. This would allow individuals to manage their privacy rights and ensure health data is used in compliance with regulations. Consent tracking involves documenting when and how individuals provide consent for the use of their data as well as specifying the purposes, scope, and duration for which their information may be accessed. Therefore, an effective consent management system enabling consent changes and revocation is essential and could be realized through a platform-based approach, like the SHC^21^. Consent tracking must interact with techniques like pseudonymization, which replaces identification data with unique codes, and anonymization (e.g., through categorization of data, adding noise to data, shifting dates, etc.), which removes and encrypts identifiers, to allow traceable and responsible data uses. Both methods protect patient identities in different ways, with pseudonymization allowing for possible reidentification if needed and anonymization rendering routine reidentification impossible (although unauthorized reidentification based on data characteristics is still possible in some circumstances^37,38^). Tracking the use of PG-HD includes insights on the types of data (e.g. heart rate, sleep patterns), the data utilization (e.g. clinical research), and the entities which the data is shared with (e.g. researchers, HCPs). Together, consent tracking and data use monitoring could not only enhance privacy but also foster trust in healthcare and research by assuring individuals that their information is securely and responsibly managed and its sharing provides real societal value.

However, despite the promise of technologies like blockchain and SSI, real-world adoption faces structural barriers. Many wearable manufacturers retain extensive rights over user data through service agreements and often monetize this data, as seen with Fitbit requiring Google accounts for access. These commercial models offer little incentive to adopt user-controlled data frameworks. As a result, implementation of transparent consent systems will likely depend on regulatory mandates, public pressure, or integration into public-sector platforms where user agency and data ethics are prioritized.

### Technologies for health data use and consent tracking

Several technologies could enable the tracking of health data use, data sharing consent, and reporting information of subsequent PG-HD uses to individuals. We therefore assess three of these technologies as having great potential for this use case: blockchain including smart contracts, self-sovereign identity (SSI) and decentralized identifiers (DIDs) as well as de-identified tokens (Table 2).

**Table 2:** Overview of the three technologies Blockchain, Self-sovereign identity/Decentralized identifiers (SSI/DIDs) and De-identified tokens on how they can be used in tracking of health data and data sharing consent

Blockchain	•Decentralized, immutable ledger technology that records transactions securely and transparently •Allows secure storage and tracking of health data use, maintaining a tamper-proof audit trail for consent and data sharing
SSI/DIDs	•A framework enabling individuals to own and control their digital identity without relying on centralized authorities •Enables individuals to manage their health data access and consent, ensuring privacy and control
De-identified tokens	•Cryptographic tokens that represent de-identified or data points for linkage of data from multiple sources •Allows linkage of health data without exposing personal identifiable information to generate comprehensive data sets

### Blockchain technology for tracking of health data use and data sharing consent

Blockchain is a decentralized, distributed digital ledger that records transactions across multiple nodes in a secure and tamper-resistant manner. It was first introduced in 2008 as the underlying technology for Bitcoin^39^. It has since expanded to various applications beyond cryptocurrencies. Each block in the chain contains transaction data, a timestamp, and a cryptographic hash of the previous block, ensuring immutability and transparency. This structure eliminates the need for a central authority, as participants in the network can independently verify transactions^40,41^. Key features of blockchain include decentralization, transparency, and enhanced security through cryptographic methods. It operates on a peer-to-peer network, making it resistant to tampering and fraud.

While widely used in finance, blockchain also holds potential in healthcare^42^. PG-HD would provide a valuable source of real-world data for healthcare and research institutions. At the same time, the risks of unauthorized sharing, data breaches, and unethical practices can undermine trust. Blockchain’s decentralization, data integrity, and lack of intermediaries offer a trust-centered infrastructure for secure information sharing and access control. By combining a consent management platform, like the SHC, with blockchain, individuals would be empowered to set their consent preferences as well as to track consent and the use of PG-HD, addressing the need for secure, personally-controlled data systems.

In healthcare, especially for tracking PG-HD and consent from DHTs, a consortium blockchain is the best option among the three different types of blockchains (Table 3). Public blockchains ensure decentralization but lack privacy, while private blockchains offer privacy but not shared control. Consortium blockchains balance both by allowing only trusted entities, like health systems, to validate transactions. This ensures privacy, accountability, and real-time tracking of PG-HD use and consent, making updates instantly visible to authorized entities. They also support traceability through a shared ledger, simplifying audits while maintaining data privacy. Once recorded, consent cannot be altered since changes are added as new blocks. This decentralized network ensures transparency, secure storage, and accurate data management by verifying transactions across multiple nodes and preserving data integrity.

**Table 3:** Overview of the three different types of blockchains

Feature	Public blockchain	Private blockchain	Consortium blockchain
Access	Open to anyone	Restricted to specific users	Limited to a group of organizations
Consensus Mechanism	Open participation (Proof of Work or Proof of Stake)	Controlled by one entity	Selected participants (Proof of Authority)
Transparency	Fully transparent	Data is private and not publicly accessible	Partially transparent among members
Control	Decentralized control	Centralized control by a single entity	Shared control among consortium members
Use Cases	Cryptocurrencies (e.g. Bitcoin^39^ and Ethereum^75^), public applications	Enterprise solutions, sensitive data	Collaborative projects, inter-organizational applications

**Fig. 1:**
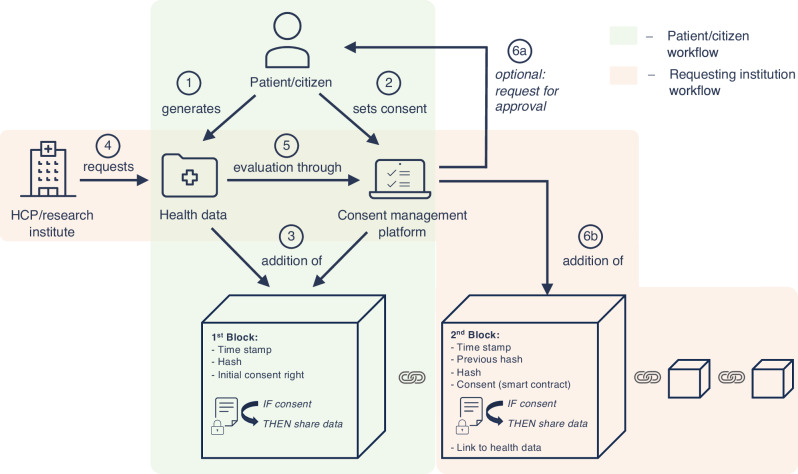
Blockchain concept for tracking of the use of personally-generated health data through apps and wearables and data sharing consent using smart contracts. The patient/citizen workflow is highlighted in green and the workflow of the data requesting institution (e.g. HCP, research institute) is highlighted in orange.

In addition to the timestamp and the hash, which ensure the linking of the blocks, the blockchain contains a link to the externally stored PG-HD as well as the consent to share this data, which is realized by smart contracts (Fig. 1).

### Smart contract managed data sharing consent

Smart contracts on the blockchain can be a useful addition to streamline complex healthcare processes by automating data-sharing agreements, payments, and various workflows. A smart contract is a software program that runs on a blockchain, capable of reading other contracts, implementing decisions, and executing contracts^43^. It can be used to store digital assets into the blockchain and claim ownership of the assets. They can create intelligent representations of the health data to which consent has been given and which is stored on individual nodes within the network and they may include metadata related to record ownership and access permissions^43^. Under EU law, smart contracts are recognized as legally significant digital agreements, but their enforceability and legal status depend on compliance with existing regulations, such as the eIDAS Regulation, which ensures the validity of electronic signatures and documents^44^.

An individual generates health data through an app or wearable (Fig. 1, step 1) holding the right on this data, which means that the individual person itself is allowed to use its data. When the individual sets its preferred consent options through a consent management platform (Fig. 1, step 2), like the SHC, this initial right is represented in a smart contract on the first block of the blockchain (Fig. 1, step 3). This initial right can then be transferred within the blockchain in order to share PG-HD. When an organization (e.g. HCP or research institute) requests access to health data (Fig. 1, step 4), this request will be evaluated through the consent management platform based on an individual’s registered preferences (Fig. 1, step 5). Either the individual (or possibly a trusted data intermediary^45^) is contacted before each sharing of their PG-HD and asked for confirmation (Fig. 1, step 6a) or the data will be shared automatically according to the set consent preferences (Fig. 1, step 6b). This generates a smart contract on the blockchain and, based on the smart contracts’ conditions, the requesting organization is granted access to the health data for the agreed period. The actual PG-HD is not stored on the blockchain, but the respective block contains a link to the data. The data requesting organization retrieves the data using encrypted access links or secure tokens, keeping the actual data off-chain for privacy. The individual (or the trusted data intermediary) can revoke consent through the consent management platform at any time, and the smart contract automatically updates the access status on the blockchain, revoking the organizations access almost immediately. The blockchain provides a complete, time-stamped history of consent grants, revocations and data access without seeing the actual PG-HD, ensuring compliance while maintaining privacy, as it only stores the access meta-data.

To enable practical integration with existing health data platforms, the SHC must interface with electronic health record (EHR) systems through secure APIs and interoperability standards such as HL7 FHIR (Supplementary Table 1). In Germany, the SHC will be connected with the identification provider network into which all health insurance companies are included. This would allow consent states to be read and respected by clinical systems in real time. When consent is updated on the SHC platform, these changes are propagated via standardized interfaces to connected platforms, ensuring that access permissions are synchronized across decentralized and centralized infrastructures. Such interoperability ensures that consent decisions are consistently enforced across clinical and research systems, enabling trusted and scalable data sharing. In this way, our platform validates the user existence and connects the local (hospital/study) identifier with the Germany-wide unique insurance identifier, while all captured consent information is managed in an interoperable (FHIR) format. Captured medical data (and consent information) is reused in the EHDS as well. Organizational structures are currently being established, such as the Research Data Center of the German Federal Health Ministry as one of the Health Data Access Bodies in Germany, that takes care about governance and data provision processes for captured medical data. This could be an example for other countries.

### Benefits, limitations and challenges of blockchain tracked health data use and smart contract managed data sharing consent

Blockchain technology enhances health data transparency by ensuring immutable records and granting individuals control over data access and usage. This transparency empowers patients, increases confidence in data sharing, and aligns with legal requirements for informed and revocable consent. From a HCP’s perspective, blockchain can reduce administrative overhead and support regulatory compliance through automated, auditable trails of consent. For researchers and society, it promotes greater access to real-world data under trustworthy conditions, enabling scalable data sharing that supports innovation.

The implementation of blockchain shows promise for tracking of PG-HD use and consent and enables innovative approaches, including “demonstrated consent,” where biological samples are linked to non-fungible tokens (NFTs, Supplementary Table 1) on a blockchain^46^. A customized large language model (LLM) presents research updates in an interactive format^46^. Various projects leverage blockchain for health data, such as RightsHash for clinical trial consent^47^, Aimedis for patient data management^48^, and MedChain^49^ for secure data sharing.

Challenges include maintaining fast transaction speeds with low energy use, addressed by permissioned blockchains using Proof of Authority, which limits consensus formation to selected participants^50^. Furthermore, ensuring interoperability with healthcare systems requires standardized protocols and APIs. Usability remains a concern, as managing private keys and complex permissions can limit accessibility for some users. Compliance with regulations like GDPR is managed by storing personally identifiable data off-chain, but outdated links could hinder access to critical data.

In blockchain-based consent management models (e.g. SSI), revocation of consent depends on recording changes directly onto the blockchain. In federated international systems, such as the EHDS, consent revocation could be managed using permissioned blockchain networks, jointly governed by HCPs or regulatory authorities from participating countries. Revocations become visible to stakeholders across borders within the timeframe of adding one new block. Additionally, off-chain channels can supplement blockchain notifications, providing redundancy and resilience during network disruptions.

Smart contracts streamline consent management by automating enforcement and reducing administrative burdens while enhancing transparency. Several pilot projects have explored the feasibility of smart contracts in health data management. For instance, MIT’s MedRec^51^ used Ethereum to manage access to electronic medical records, while the EU-funded MyHealthMyData^52^ project tested blockchain-based consent mechanisms for sharing health data. These pilots show that smart contracts can technically enforce consent preferences, though real-world adoption depends on integration with healthcare systems and user-friendly interfaces.

However, they must adapt to evolving regulations and handle complex consent scenarios. Their rigidity can create issues when legal requirements change, and third-party oracles that connect blockchains to external data sources, enabling smart contracts to interact with off-chain data introduce potential security risks^53^. Mitigation strategies include multi-oracle systems^54^ and consensus mechanisms, but regulatory challenges remain a barrier to their broader adoption in healthcare.

### Self-Sovereign Identity (SSI) and Decentralized Identifiers (DIDs)

A useful addition to blockchain that could robustly and securely inform citizens on uses of their PG-HD is self-sovereign identity (SSI). SSI is a digital identity approach that allows individuals to control the information they use to verify their identity with websites, services, and applications across the internet. Without SSI, users who want to maintain consistent identities online often depend on major identity providers like Facebook (Facebook Connect) and Google (Google Sign-In), which manage and control their identity information^55^. For those who prefer not to use these providers, the alternative is to create separate accounts for each service, leading to a fragmented online experience. With SSI, users control and own their digital identities and other verifiable digital credentials (VCs) without having to rely on a central organization such as Facebook or Google. SSI provides a solution to both issues by enabling users to access services securely and seamlessly, while retaining full control over their identity information.

Unlike traditional identity systems managed by central authorities, self-sovereign identity (SSI) is user-centered, giving individuals full control over their digital identity and data, including consent for health data sharing. SSI uses World Wide Web Consortium standards like decentralized identifiers (DIDs) and the VC Data Model, which leverage blockchain for secure credentials and identity data^56^. DIDs are decentralized, self-managed, and globally unique, enabling users to control and create multiple identifiers. Health platforms could link data and consent to DIDs, making consent portable and interoperable across health systems and organizations.

**Fig. 2:**
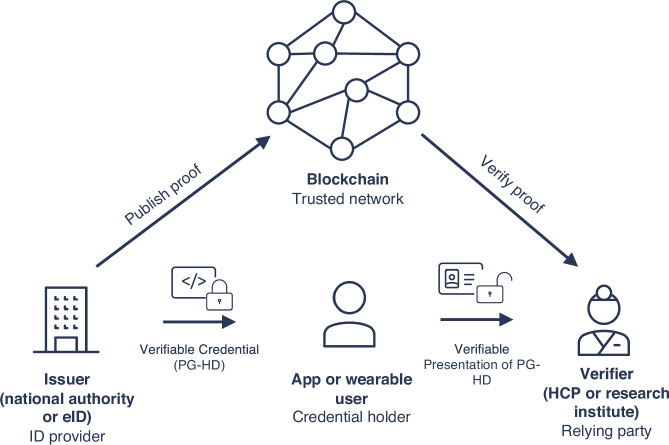
Schematic representation of using Self-Sovereign Identity and Decentralized Identifiers to track health data use and data sharing consent. A secure process is initiated when the user presents ist verifiable credentials, enabling the verifier to scan it, establish a private channel, and receive verifiable credential data for authentication.

The creation of DIDs could be possible through two options: either the DID is generated by a national authority or the DID is created by the individual themselves. However, in this case, authorization is required to ensure that the generated DID truly belongs to the person and to prevent the creation of so-called “fake accounts”. This authorization could be realized through electronic identities (eIDs, Supplementary Table 1), for example the European Digital Identity provided by the European Commission, which can be used by European citizens to identify themselves or provide confirmation of certain personal information^57^. Each individual is assigned a DID, which they control through cryptographic keys stored in their digital wallet. The DID acts as a persistent, unique identifier for the individual but does not inherently reveal personal information. Health data, such as PG-HD tracked by an app or wearable, can be issued by the HCP as VCs linked to the individuals DID (Fig. 2). These VCs can be selectively disclosed by the individual when needed, e.g. sharing only specific data with research institutions. Similarly, individuals can also issue and manage consent through their DID. A consent record is generated as a VC, which specifies the data that can be accessed, the purpose of access and the duration of the consent (Fig. 2). These consent credentials are stored in the individuals’ wallet or a consent management platform, like the SHC, and can be revoked or updated by the individual at any time. The actual health data is stored off-chain while access rights are managed on-chain using blockchain and smart contracts to ensure transparency and immutability. This serves as a trust layer, acting as a verifiable data registry to securely and reliably store public keys, among other functions, and providing cryptographic protection as an anonymous revocation registry. In this way, the tracking of the lifecycle of each consent instance, from issuance to renewal, modification, or revocation in a decentralized, tamper-resistant ledger is possible and provides an immutable history of consent actions. When a patient wants to revoke or update consent, the change can be immediately reflected across the ecosystem without the need to update multiple records and fear of data leaks. This ensures up-to-date records of who has authorized access, what data is accessible, and under what terms for all stakeholders (HCPs, research institutes, insurance, etc.). The consent agreements can be embedded with expiration dates, which automatically invalidate access to health data after a certain period or event, e.g. the completion of a clinical study. The individuals can receive notifications about upcoming consent expirations via their digital wallets or platforms and decide whether to renew, modify, or revoke their consent. Automated consent management could be realized by encoding consent agreements into smart contracts linked to DIDs. These smart contracts on a blockchain could trigger specific actions upon consent revocation or expiration, like immediately notifying the HCP or the individual and blocking access to data. Additionally, individuals can receive notifications on how their health data is used, e.g. in case of a new study which includes relevant data.

PG-HD - Personally-Generated Health Data.

### Benefits, limitations and challenges of SSI and DIDs for tracking of health data use and consent

SSI and DIDs give individuals full control over the use of PG-HD and consent, while helping organizations comply with privacy regulations like GDPR and HIPAA through dynamic consent tracking and data minimization. Individuals can manage data sharing, track access logs, and revoke consent, with smart contracts ensuring regulatory compliance. For patients, this translates into the ability to manage data access, track usage through access logs, and revoke consent dynamically, thereby fostering a sense of empowerment and security^58^. From the perspective of HCPs, SSI and DIDs help ensure compliance with data protection regulations like GDPR and HIPAA by supporting fine-grained consent tracking and data minimization practices. For researchers and society, these technologies can enhance the reliability and integrity of consent processes, and facilitate ethical reuse of health data across systems.

However, a major drawback of SSI is the risk of losing access to a DID, as it could mean losing control over linked health data. To mitigate this risk, multi-key and multi-signature approaches could be used. A user can split the control across multiple devices or use threshold cryptography (e.g. requiring at least two keys to regain access). For non-expert users, practical implementations increasingly include user-friendly key recovery solutions, such as social recovery mechanisms (where trusted, predetermined contacts are given backup keys that help restore access)^59^, biometric reauthentication^60^, or encrypted backups stored via custodial agents or distributed storage networks (off-chain) or the HCP^61^. Although these solutions also have some trade-offs they can help to balance security with ease of use, while also keeping the time criticality of clinical workflows in mind. As consent for the use of PG-HD is not time critical, trusted contacts such as friends, family or doctors’ offices can be used for key recovery with no harm being caused by additional delays, where a patient can request the recovery keys from either the social entities or the custodial entities upon realizing the loss of their own keys and wait for the transmission of their recovery keys. However this would be a limitation for the use in the management of EHR access. Another drawback is the usability of SSI/DID because of its complexity for non-technical users to manage cryptographic keys, and identity credentials securely and efficiently. Furthermore, the fragmentation of health data systems and varying standards present challenges for interoperability with EHRs and health providers’ platforms. To improve this, systems should adhere to open standards like HL7 FHIR. A unified framework for data exchange and consent management, supported by collaborations among HCPs, developers, and regulators, is essential.

### Linking of health data via privacy-preserving record linkage and de-identified tokens

Privacy-preserving record linkage (PPRL) is a process that identifies records corresponding to the same real-world entities across multiple databases while ensuring that sensitive or confidential information about these entities is not disclosed during the linkage process^62^. PPRL is widely used in applications such as healthcare and public policy, where privacy regulations require secure integration of data from different sources^63,64^. Based on the PPRL approach, similar techniques have been developed and commercialized, such as de-identified tokens. De-identified tokens are generated by encrypting personally-identifiable information (PII) into unique placeholders that cannot be reverse-engineered to reveal the original information^65^. This technology aims to create the same patient-specific tokens in any data set, which means that two or more related data sets can be linked as well as tracked using the patient tokens without ever sharing the underlying patient information. In healthcare, they could potentially enable organizations to integrate and analyze patient data from various sources, such as data from apps, electronic health records, claims data, and clinical research, while ensuring privacy^66,67^. For data sharing, patients can grant consent for their de-identified information to be accessed, facilitating clinical studies without compromising their identity. This approach ensures compliance with privacy regulations while enabling the seamless flow of health information for research and healthcare coordination.

**Fig. 3:**
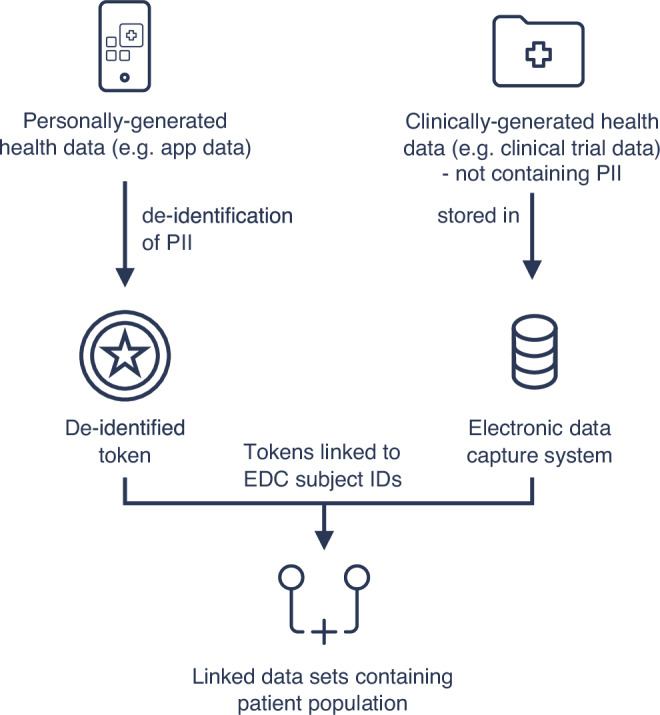
Schematic representation of using de-identified tokens to link personally-generated health data with clinically generated health data. De-identified tokens allow secure linkage of personally-generated health data with clinically generated health data through a privacy-preserving mechanism that maintains individual anonymity while enabling meaningful data integration for research and care. PII - Personally-Identifiable Information; EDC - Electronic Data Capture.

In practice, de-identified tokens can be assigned to individuals across multiple data sources which would enable linking of PG-HD from apps and wearables with clinically generated data. This could be used to bridge evidence gaps by connecting real world data (RWD, Supplementary Table 1), like apps or wearables data to clinical trials in order to understand real world outcomes^68^. The usage of RWD was also acknowledged in regulatory decision making and therefore regulators have passed key legislation and issued guidance to support its usage^69–71^. For example, when a patient agrees on the linkage of his PG-HD from a health app with clinically generated data, the patient’s PII is encrypted in a de-identified token. This token can then be linked to non-PII clinical trial data collected in an electronic data capture system and can be assessed by an HCP or research institute with a minimized risk of unblinding or re-identifying patients (Fig. 3).

De-identified tokens also play a pivotal role in managing health data sharing consent by securely linking patient preferences with their data without exposing PII. These tokens could enable consent management across systems, ensuring that requests for data access align with the patient’s consent status. Patients can dynamically update their preferences, such as opting out of certain data-sharing agreements, with the tokens ensuring these changes are accurately reflected across linked datasets. Additionally, the tokens allow for granular consent tracking, enabling patients to specify how their data can be used, for instance, allowing de-identified lab results for research but restricting identifiable clinical notes.

### Benefits, limitations and challenges of de-identified tokens for tracking of health data use and consent

De-identified tokens potentially enhance privacy as they are highly resistant to re-identification and improve interoperability for secure data linkage, benefiting medical research^67^. For patients, this provides a layer of protection against unauthorized data exposure, while still allowing their health data to contribute to research and care coordination. This privacy-preserving approach can enhance patient trust in data-sharing initiatives, particularly when tokens are used in transparent, consent-driven systems. For HCPs, de-identified tokens improve interoperability by enabling secure linkage of disparate datasets, such as EHRs, app-generated data, and research databases, without exposing PII. This supports more accurate population health analytics and coordinated care models. For researchers and society, tokenization facilitates the aggregation of rich, multi-source datasets necessary for advancing precision medicine, generating real-world evidence, while respecting data minimization principles.

However, linking datasets introduces privacy risks, particularly the potential for cross-dataset re-identification. As highlighted in recent debates in biomedical informatics, even de-identified data may contain unique patterns, such as treatment combinations, rare conditions, or longitudinal health trajectories, that could be exploited to re-identify individuals when combined across multiple datasets. This risk is especially pronounced when datasets are large, richly detailed, and linked with external sources. While such re-identification remains technically challenging, the theoretical possibility demands caution, particularly as analytic techniques and external data sources evolve. Further privacy issues can arise should a PPRL be performed in peer-to-peer settings. This can enable brute-force attacks in which a malicious party attempts to re-identify individuals in a dataset by trying to match excessive amounts of false data, which effectively lead to matches and re-identification with sufficient fake data. The tension between maximizing data utility and preserving privacy cannot be eliminated, only carefully managed. One way to mitigate brute-force attacks is to adopt a secure multi-party computation (SMPC)-based peer-to-peer approach^72^ or to involve a trusted third party (TTP)^73^ as linkage entity. While the former SMPC approach is less suited for larger datasets due to algorithmic complexity, the TTP is performant with larger datasets and can enact filters on malicious datasets, preventing unrealistic dataset sizes or matching frequencies, required for brute-force attacks. Other investigated mitigations could include minimizing the linkage between source and encoded records through zero-relationship encoding which effectively preserves privacy^74^. Furthermore, de-identified tokens can be utilized for linking PG-HD to clinically generated data in prospective data collections where control is maintained, but their applicability to retrospective data collections is limited and would need alternative approaches, such as record linkage via indirect identifiers or probabilistic matching. Limitations arise when token generation relies on incomplete or inconsistent input data, potentially leading to inaccurate linkages. Additionally, the process requires robust technological infrastructure and governance frameworks to maintain token integrity and prevent misuse. By leveraging de-identified tokens, the healthcare sector must navigate this inherent tension between data privacy and the need for comprehensive, actionable insights to improve patient care and public health outcomes.

### Comparative summary of the three technologies

Each of the three above reviewed technologies offers distinct advantages and is best suited to different implementation scenarios based on control granularity, interoperability, and user-friendliness. Blockchain and SSI/DIDs are often used together, as blockchain provides the decentralized infrastructure for identity registries and credential verification, while SSI frameworks enable fine-grained, user-managed consent through W3C-standardized verifiable credentials. This combination is well-suited when auditability, selective disclosure, and cross-organizational trust are required, such as in longitudinal care pathways or cross-border data sharing. While this layered model promotes transparency and patient autonomy, real-world deployment remains constrained by limited wallet adoption, key management complexity, and uneven interoperability with existing healthcare infrastructure. In contrast, de-identified tokens are best suited for scenarios where large-scale datasets must be linked without continuous user involvement, for instance, in retrospective research, public health monitoring, or health service planning. These methods reduce exposure of identifiable data and support scalable integration, but typically rely on coarse-grained consent and require careful governance to mitigate re-identification risks. In practice, a hybrid approach leveraging all three technologies may offer the most comprehensive solution. Blockchain with SSI/DIDs supports personalized consent and accountability, while de-identified tokens enable secure linkage across institutional silos, together facilitating privacy-preserving, interoperable health data ecosystems.

### Practical implementation barriers and resource implications

While the proposed technologies, blockchain, SSI/DIDs, and de-identified tokens, offer promising pathways for privacy-preserving and transparent data governance, practical implementation in real-world healthcare settings remains challenging. Many patients may lack the digital literacy to manage consent preferences or cryptographic credentials, and resource-limited health systems may struggle with integration and upkeep. To address this, solutions include simplified user interfaces, digital literacy training, integration of consent tools into familiar patient portals, and automation of routine tasks. Public-private partnerships and targeted funding can help resource-constrained providers adopt and sustain these technologies. Importantly, inclusive design and stakeholder co-development must ensure accessibility for digitally marginalized populations, promoting trust and equitable participation in health data sharing.

## Conclusion

**Fig. 4:**
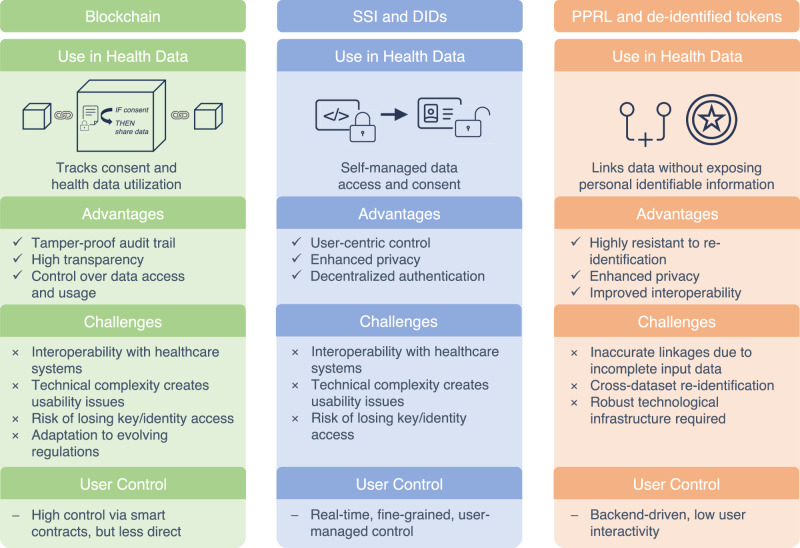
Comparison of all three technologies (Blockchain, SSI and DIDs, Privacy-preserving record linkage (PPRL) and de-identified tokens) and their use in health data management. Each technology offers unique advantages, but also presents specific challenges that must be considered in implementation.

In conclusion, secure management of health data and consent is essential for safeguarding autonomy and advancing healthcare research. Trust and transparency play a critical role in encouraging data sharing, which enables access to RWD, benefiting both individual and societal health. Emerging technologies like blockchain, SSI, DIDs, and de-identified tokens offer innovative solutions to modernize tracking of health data use and consent and enhance transparency (Fig. 4). Linking these technologies with platforms like the SHC empower individuals to control their data and consent actively, fostering trust and increasing participation in data-sharing initiatives. Integrating these technologies with healthcare systems will be crucial to address legal and technical challenges, ensuring responsible and secure data use. As a next step, technical prototyping should be pursued to evaluate integration with existing health IT infrastructure and ongoing prototypes such as Gaia-X, TEHDAS 2, or national prototypes for the EHDS such as Germany’s “Health Data Access Body”, accompanied by stakeholder validation, including patients, HCPs, developers and regulators, to assess usability and trust.

## Supplementary information

Supplementary Information

## Data Availability

No datasets were generated or analysed during the current study.
