# Supplementary material for: Enabling secure and self determined health data sharing and consent management
Source: NPJ Digit Med. 2025 Aug 30;8:560. doi: 10.1038/s41746-025-01945-z (PMC12398500; doi:10.1038/s41746-025-01945-z)
Supplement: Supplementary file 1 — Supplementary Information [file 41746_2025_1945_MOESM1_ESM.pdf]

Supplementary Material:

Supplementary Table 1: Glossary of Terms and Acronyms

**Blockchain**	A decentralized and immutable digital ledger that securely records transactions across a distributed network. It enhances transparency, traceability, and trust in data management.
**Smart Contract**	A self-executing program on a blockchain that automatically enforces agreed rules and conditions (e.g., data access or consent preferences) without needing intermediaries.
**Self-Sovereign Identity (SSI)**	A decentralized identity model that allows individuals to fully control their digital identity and credentials without relying on a central authority.
**Decentralized Identifier (DID)**	A globally unique identifier that is created and controlled by an individual, not tied to a central registry or authority. Used in SSI systems to represent and manage identity securely.
**De-identified Token**	A cryptographically generated placeholder that represents a person in a dataset without revealing their identity, enabling linkage of records across systems while preserving privacy.
**Privacy-Preserving Record Linkage (PPRL)**	A method that connects data from different sources about the same individual without disclosing personal identifiers, ensuring privacy during data linkage.
**Personally-Generated Health Data (PG-HD)**	Health-related data actively or passively created by individuals, often through digital health tools (e.g., apps, wearables), outside traditional clinical settings.
**Consent Management Platform (e.g., SHC)**	A digital system that allows individuals to provide, update, and revoke consent for the use of their personal data, ensuring transparency and regulatory compliance.
**Standard Health Consent (SHC)**	A proposed platform-based consent model that standardizes how individuals give permission for their health data to be collected and shared, combining features of broad, dynamic, and meta consent.
**Dynamic Consent**	A flexible, ongoing consent model allowing individuals to give or withdraw consent for each use of their data, typically through an app or online interface.
**Broad Consent**	A consent model in which individuals give permission for the future use of their data for unspecified research within a general scope, often with an option to withdraw later.
**Meta Consent**	A hybrid model allowing individuals to choose how they want to provide consent—broad or dynamic—depending on the type of data use or study.
**Electronic Health Record (EHR)**	A digital version of a patient’s paper chart, including clinical data managed by a healthcare provider.
**European Health Data Space (EHDS)**	An EU initiative aimed at enabling secure exchange and use of health data across Europe for healthcare delivery, research, and policy-making.
**HL7 FHIR (Fast Healthcare Interoperability Resources)**	A standard describing data formats and APIs for exchanging electronic health records, widely used to ensure interoperability in health IT systems.

**General Data Protection Regulation (GDPR)**	The European Union regulation governing data protection and privacy, particularly for personal data, including health-related data.
**Non-Fungible Token (NFT)**	A unique digital asset recorded on a blockchain, often used to represent ownership of a specific item—such as a biological sample linked to consent.
**Real-World Data (RWD)**	Data relating to patient health or healthcare delivery routinely collected from a variety of sources outside of traditional clinical trials.
**Electronic Identity (eID)**	A government-issued digital identity that can be used to authenticate individuals online for accessing secure services and data.
